# Recurrent pain in a child with cerebral palsy: Questions

**DOI:** 10.1007/s00467-021-05147-z

**Published:** 2021-07-29

**Authors:** Andrea Trombetta, Simone Benvenuto, Egidio Barbi

**Affiliations:** 1 University of Trieste, Trieste, Italy; 2 Institute for Maternal and Child Health – IRCCS “Burlo Garofolo”, Trieste, Italy

**Keywords:** Child, Abdominal pain, Cerebral palsy

## Case presentation

**Fig. 1:**
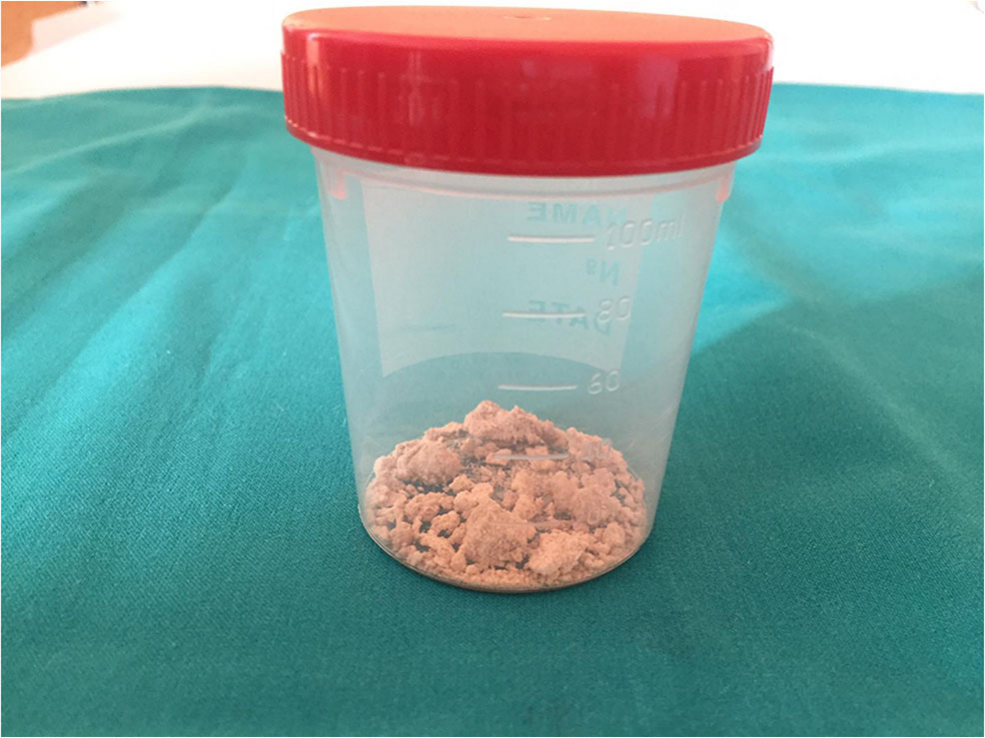
Brownish aggregates found in the child’s diaper

An eight-year-old child was admitted for a 5-week history of recurrent abdominal pain. The pain was reported to be present almost daily, disrupting sleep and causing episodes of agitation and restlessness, with sudden and incessant crying and increased heart rate, particularly when the child was moved from the stroller to his bed. His mother reported a partial improvement after ibuprofen administration. His previous history was remarkable for developmental delay, cerebral palsy with spastic tetraparesis, drug-refractory epilepsy treated with vigabatrin, levetiracetam, topiramate, and phenobarbital, and the placement of a percutaneous endoscopic gastrostomy 6 years earlier. The parents did not report any recent history of vomiting, diarrhea, constipation, or fever. A dental evaluation ruled out tooth decay, and an orthopedic evaluation excluded an osteoarticular issue. Laboratory tests were performed to investigate possible causes of pain, including white blood cell count, glucose, alanine aminotransferase, aspartate aminotransferase, creatinine, electrolytes, bilirubin, amylase, and lipase, and they were all in the normal range (Table 1). Physical examination was unremarkable, but brownish aggregates were noted when the diaper was removed (Fig. 1).

**Table 1:** Laboratory tests

Laboratory test	Value (reference range)
Total WBC (cells/μL)	12110 (6000–13,000)
Neutrophils (cells/μL)	7050 (1500–8500)
Lymphocytes (cells/μL)	3980 (2000–8000)
Monocytes (cells/μL)	730 (10–1000)
Eosinophils (cells/μL)	330 (100–500)
Basophils (cells/μL)	20 (0–20)
Creatinine (mg/dL)	0.29 (0.2–1.15)
BUN (mg/dL)	10 (6–21)
CRP (mg/dL)	4.2 (< 5.0)
ALT (U/L)	3 (10–25)
AST (U/L)	23 (23–46)
Bilirubin (mg/dL)	0.4 (0.2–1)
GGT (U/L)	12 (5–14)
Glucose (mg/dL)	76 (55–105)
Na^+^ (mEq/L)	144 (135–145)
K^+^ (mEq/L)	3.5 (3–5.5)
Ca^2+^ (mg/dL)	9.2 (8.8–10.8)
Mg^2+^ (mg/dL)	1.6 (1.4–1.7)
Amylase (U/L)	127 (98–405)
Lipase (U/L)	23 (5–160)

*WBC* white blood cell, *BUN* blood urea nitrogen, *CRP* C-reactive protein, *ALT* alanine aminotransferase, *AST* aspartate aminotransferase, *GGT* γ-glutamyltransferase

## Questions

1. What is the most likely cause of this child’s pain?

2. How should the diagnostic workup be completed?

3. What are the best treatment and follow-up for this patient?

## Data Availability

Not applicable.

